# Delirium Occurrence and Associated Factors Among Patients With Dementia and Mild Cognitive Impairment Admitted to Long-Term Care Hospitals: Cross-Sectional Study

**DOI:** 10.2196/90434

**Published:** 2026-10-27

**Authors:** Kyoung Ja Moon, Yong-Hwan Hyeon

**Affiliations:** 1 Department of Nursing College of Nursing Keimyung University Daegu Republic of Korea; 2 Department of Nursing Ulsan College of Nursing Ulsan College Ulsan Republic of Korea

**Keywords:** delirium, dementia, mild cognitive impairment, long-term care, cross-sectional study

## Abstract

**Background:**

Delirium is common among older adults, particularly those with cognitive impairment. However, delirium among patients with dementia or mild cognitive impairment (MCI) in long-term care (LTC) hospitals has received limited attention.

**Objective:**

This study aimed to examine delirium occurrence and associated factors among patients with dementia or MCI in LTC hospitals.

**Methods:**

This cross-sectional study included 142 patients aged 65 years or older with dementia (n=100, 70.4%) or MCI (n=42, 29.6%) at 2 LTC hospitals in South Korea between September 2021 and August 2022. Delirium occurrence was defined as at least 1 positive Short Confusion Assessment Method assessment during twice-daily assessments over 4 weeks, including the first assessment. Multivariable logistic regression examined associated factors overall and within each cognitive group.

**Results:**

Delirium was identified in 55 (38.7%) of 142 patients. The proportion was 41% (41/100) in patients with dementia and 33.3% (14/42) in patients with MCI. Hypoactive delirium was the most common subtype (33/55, 60%). Overall, female sex (adjusted odds ratio [aOR] 4.344, 95% CI 1.794-10.515; *P*=.001), immobility (aOR 4.953, 95% CI 1.857-13.205; *P*=.001), and analgesic use (aOR 3.641, 95% CI 1.262-10.501; *P*=.02) were associated with delirium. Infection was inversely associated with delirium (aOR 0.251, 95% CI 0.101-0.623; *P*=.003). In patients with dementia, female sex, immobility, analgesic use, and pain were associated with delirium. Diaper use was associated with delirium in patients with MCI.

**Conclusions:**

Delirium was common among patients with dementia or MCI in LTC hospitals. The findings support regular delirium assessment, with attention to mobility and pain-related care needs. Further studies are needed to clarify the factors associated with delirium in each cognitive group.

## Introduction

Delirium is an acute disturbance in attention, awareness, and cognition that fluctuates over time [1]. Delirium occurs in 21% of patients after hip fracture surgery [2], 48% of patients during follow-up in long-term care (LTC) facilities [3], and 24.6% of patients on admission to acute hospitals [4]. Among hospitalized older adults with dementia, the prevalence of delirium is 48.9% [5]. Although these proportions differ by population and assessment period, delirium is common across care settings. Delirium is associated with cognitive decline [6], increased mortality, longer hospital stays, institutionalization, higher health care costs, and increased caregiver burden [7-9]. Early recognition is particularly important for older patients with cognitive impairment, whose cognitive and functional difficulties may complicate delirium assessment and care.

Early identification of patients at risk is important for delirium prevention and management [5,7]. Delirium risk is related to both underlying vulnerability and acute clinical stressors [10]. Assessment therefore needs to consider cognitive status, physical illness, medication use, and daily care needs. Multicomponent nonpharmacological interventions can help prevent delirium in hospitalized patients outside intensive care units (ICUs) [11]. However, evidence for preventive interventions in institutional LTC remains limited [12]. Differences in patient characteristics and care needs require attention when applying findings from acute hospitals to LTC hospitals. Identifying factors associated with delirium in LTC hospitals can help nurses assess patients at risk and develop appropriate preventive care.

Most delirium research has focused on patients in ICUs, acute hospitals, and surgical settings. Delirium is also common among patients with dementia in LTC hospitals [3]. Patients with dementia who experience delirium have longer hospital stays and poorer outcomes than those without delirium [5,13]. Changes in attention, cognition, and behavior may be difficult to distinguish from existing dementia symptoms, making comparison with the patient’s usual condition important [13]. In residential care, delirium prevalence ranges from 1% to 70.3%, with the highest proportion in a population with dementia. Cognitive impairment and dementia are consistently associated with delirium in these settings [14]. However, the wide variation in patient populations and assessment periods limits direct comparisons across studies.

Patients with mild cognitive impairment (MCI) also require attention in delirium care. MCI is associated with progression to dementia, although the rate varies by population, diagnostic criteria, and follow-up period [15]. Physical inactivity, smoking, diabetes, obesity, and hypertension are modifiable factors associated with dementia [16]. Dementia also places a substantial financial burden on care in South Korea [17]. These clinical and care burdens support the need to address cognitive health in patients with MCI as well as those with dementia. However, the clinical and care-related factors associated with delirium in patients with MCI in LTC hospitals remain unclear. Examining both groups may help identify their delirium care needs.

Older patients in LTC hospitals often have chronic illnesses and cognitive impairment that require ongoing nursing care. Nurses observe changes in patients’ condition during daily care and have an important role in recognizing possible delirium. Information on delirium occurrence, motor subtypes, and associated factors can help nurses identify patients who need further assessment. Findings from patients with dementia and those with MCI may also help guide care according to cognitive status. However, evidence specific to these patients in LTC hospitals is needed to support delirium assessment and management.

This study examined delirium occurrence, motor subtypes, and associated factors among patients with dementia or MCI in LTC hospitals. Factors associated with delirium were examined in all participants and in the dementia and MCI groups. The findings may help guide nursing assessment and delirium care for patients with cognitive impairment in LTC hospitals.

## Methods

### Study Design

This observational study used a cross-sectional analysis of patient-level data collected through repeated assessments over 4 weeks. Delirium occurrence was defined as at least 1 positive Short Confusion Assessment Method (S-CAM) assessment during this period, including the first assessment. Each patient was classified once as having or not having delirium, regardless of the number of positive assessments. New episodes after a delirium-free baseline were not analyzed separately. The proportion of patients with delirium therefore represents the observed 4-week period prevalence, not incidence. The study followed the STROBE (Strengthening the Reporting of Observational Studies in Epidemiology) guideline for cross-sectional studies [18] (Multimedia Appendix 1).

### Participants and Recruitment

The minimum sample size was 136 patients, calculated using G*Power (version 3.1.9; Heinrich-Heine-University Düsseldorf) for multiple linear regression (*f*²=0.15, α=.05, power=0.90, and 8 predictors).

Participants were patients aged 65 years or older at 2 LTC hospitals in cities D and K, South Korea, between September 2021 and August 2022. Each hospital had approximately 250 to 300 beds and mainly cared for older patients, including those with dementia. Patients were eligible if they had a physician-recorded diagnosis of dementia or MCI. After approval from the directors of nursing, recruitment notices were posted on ward notice boards. Head nurses received 2 training sessions and served as research assistants. They checked eligibility and explained the study individually using the recruitment notice and participant information sheet. Patients who expressed interest after reading the notice were also considered. Written consent was obtained from patients or their legal guardians. Of 168 patients assessed, 18 (10.7%) met the exclusion criteria, and 8 (4.8%) declined participation. The remaining 142 (84.5%) patients were included in the analysis (Figure 1).

**Figure 1:**
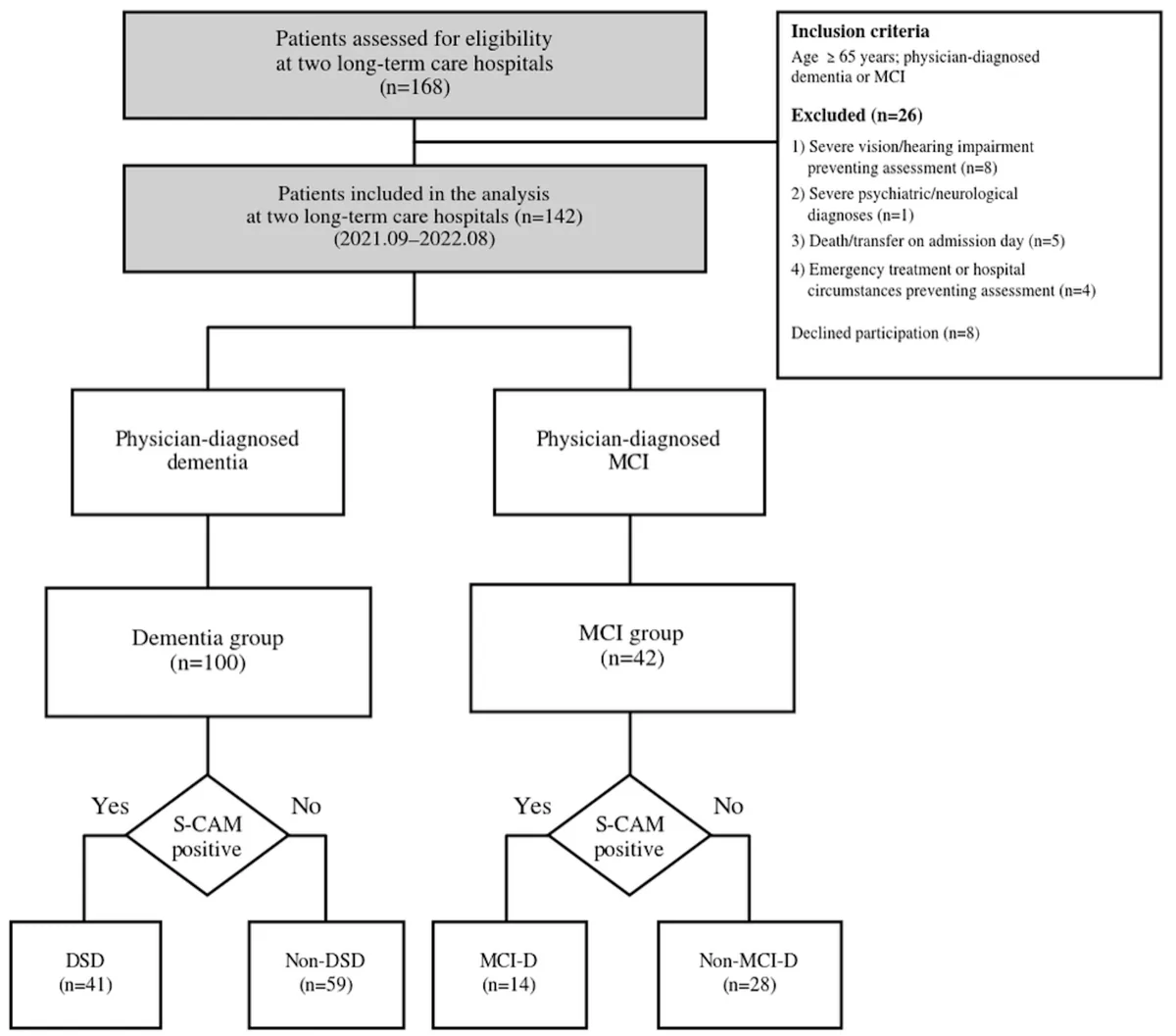
Recruitment and classification in this cross-sectional study. DSD: delirium superimposed on dementia; MCI: mild cognitive impairment; MCI-D: MCI with delirium; non-DSD: dementia without delirium; non-MCI-D: MCI without delirium.

Patients were excluded because of severe visual or hearing impairment that prevented S-CAM assessment (n=8), severe psychiatric or neurological diagnoses (n=1), death or transfer on the day of admission (n=5), or inability to undergo assessment because of emergency treatment or hospital circumstances (n=4).

Patients were classified into the dementia group (n=100, 70.4%) or the MCI group (n=42, 29.6%) according to their physician-recorded diagnosis. The Korean version of the Mini-Mental State Examination (K-MMSE) cutoffs were not used for eligibility or group assignment. The groups were further divided according to whether patients had any positive S-CAM assessment during the 4 weeks (Figure 1): (1) patients with dementia and at least 1 positive S-CAM assessment (delirium superimposed on dementia [DSD]; n=41), (2) patients with dementia and no positive S-CAM assessment during the observation period (dementia without delirium [non-DSD]; n=59), (3) patients with MCI and at least 1 positive S-CAM assessment (MCI with delirium [MCI-D]; n=14), and (4) patients with MCI and no positive S-CAM assessment during the observation period (MCI without delirium [non-MCI-D]; n=28).

### Measures

#### General and Clinical Characteristics

Baseline characteristics included age, sex, admission source, Charlson Comorbidity Index (CCI) score, BMI (kg/m²), K-MMSE score, number of medications, smoking, alcohol consumption, and comorbidities. The K-MMSE [19] score was used as a continuous measure of cognitive status rather than to classify patients as having dementia or MCI.

Clinical and care-related variables recorded during the 4 weeks included visual or hearing impairment, dehydration (blood urea nitrogen >18 mg/dL), malnutrition, fluid and electrolyte imbalance, infection, pain, sleep deprivation, physical restraint use, immobility, recent surgery, falls, pressure ulcers, analgesic use, blood transfusion, urinary catheterization, enteral feeding, diaper use, and sedative use. Haloperidol was used only in patients with delirium and was not included in the regression models. A clinical condition was coded as present if it was recorded at least once during the assessment period. Infection was identified through a physician diagnosis, laboratory evidence, or antibiotic use. Immobility was assessed through routine activities-of-daily-living assessments and observation. Pain was assessed using recorded pain scores and pain medication use. Analgesic and sedative use were recorded as yes or no variables.

#### Delirium Assessment and Classification

Delirium was assessed using the S-CAM, based on the assessment guidelines [20] and previous research [3]. The S-CAM includes acute onset and fluctuating course, inattention, disorganized thinking, and altered level of consciousness. A positive result requires the presence of the first 2 features, along with either disorganized thinking or an altered level of consciousness. Nurses identified acute changes by comparing patients’ current condition with their usual cognitive and behavioral status. Motor subtypes were classified as hypoactive or hyperactive according to the main observed pattern. Patients who alternated between hypoactive and hyperactive features were classified as having mixed delirium.

Delirium was assessed twice daily for 4 weeks, in the morning (8-10 AM) and afternoon (4-6 PM). Head nurses served as research assistants, and the nurse in charge of the participating unit performed the afternoon assessments. Before data collection, head nurses received 2 training sessions. A head nurse and a researcher then assessed the same 5 to 6 patients at the same time and compared their checklist ratings. Training continued until an agreement rate of approximately 98%-99% was achieved.

### Statistical Analysis

Data were analyzed using SPSS (version 23.0; IBM Corp). Descriptive statistics summarized participant characteristics and delirium occurrence. The proportion with delirium was calculated as the number of patients with at least 1 positive S-CAM assessment during 4 weeks divided by the number analyzed (142 overall, 100 with dementia, and 42 with MCI), multiplied by 100. Group differences were examined using 1-way ANOVA, Pearson chi-square, or Fisher-Freeman-Halton exact tests. Fisher exact tests compared delirium occurrence and motor subtypes between cognitive groups. All tests were 2-sided (*P*<.05). No analyzed values were missing.

Multivariable logistic regression examined factors associated with delirium in the overall sample and separately within the dementia and MCI groups. The dependent variable was delirium occurrence, coded as at least 1 positive S-CAM assessment during 4 weeks vs none. The overall model included age, sex, CCI score, K-MMSE score, infection, immobility, and analgesic use. Subgroup variables were selected using univariable *P*<.05, previous research, and clinical relevance. Variables were entered simultaneously, with male sex and absence of binary characteristics as references. Adjusted odds ratios (aORs) and 95% CIs were reported. Each aOR describes the association between a factor and delirium within the analyzed sample, conditional on the other variables in that model. Multicollinearity and model fit were assessed using variance inflation factors and the Hosmer-Lemeshow test.

### Ethical Considerations

The study was approved by the Institutional Review Board of Keimyung University (IRB 40525-202012-HR-073-06) and was conducted in accordance with the Declaration of Helsinki. Written informed consent was obtained from patients or their legal guardians. Paper consent and data collection forms were stored in a locked cabinet and later destroyed. Anonymized electronic data are retained in encrypted form. The approved plan specified data retention for 3 years after study completion. Participants received a cotton T-shirt worth approximately ₩10,000 (₩1=US $0.000757 as of August 2022) as compensation.

## Results

### Participant Characteristics

Of 168 patients assessed for eligibility, 18 met exclusion criteria, and 8 declined participation. The analysis included 142 patients, whose mean age was 80.92 (SD 7.26) years; 58.5% (n=83) were female, and 41.5% (n=59) were male. Smokers accounted for 2.1% (n=3) of patients, and none reported alcohol consumption. Most patients (n=93, 65.5%) were admitted from home.

The mean BMI was 21.97 (SD 3.44) kg/m^2^, the mean CCI score was 5.54 (SD 1.81), and the mean K-MMSE score was 10.89 (SD 7.35). Participants were taking an average of 7.50 (SD 4.81) medications. Of the 142 participants, hypertension, diabetes, and degenerative arthritis were present in 53.5% (n=76), 26.1% (n=37), and 2.1% (n=3), respectively. In addition, 54.2% (n=77) had cancer, and 1.4% (n=2) had a history of COVID-19. A history of stroke was recorded in 18.3% (n=26) of patients and a history of brain injury in 10.6% (n=15). Moreover, 1 (0.7%) patient had a visual or hearing impairment.

Among the 142 participants, 4.9% (n=7) had a blood urea nitrogen level above 18 mg/dL, 39.4% (n=56) had malnutrition, and 26.8% (n=38) had a fluid or electrolyte imbalance. A postoperative state was recorded in 7.7% (n=11) of patients, infection in 39.4% (n=56), and pain in 19.7% (n=28). Postoperative state referred to surgery performed at another hospital before admission to the LTC facility and before the delirium assessment period. The maximum interval since surgery and the type of procedure were not recorded.

Sleep deprivation was recorded in 8.5% (n=12) of patients and physical restraint use in 3.5% (n=5). Immobility was recorded in 25.4% (n=36) of patients, a history of falls in 18.3% (n=26), and pressure ulcers in 16.9% (n=24). Analgesics were used by 16.2% (n=23) of patients. Haloperidol was administered to 14 (9.9%) patients: 6 with DSD and 8 with MCI-D. Blood transfusions were recorded in 0.7% (n=1).

Of the 142 participants, urinary catheters were used by 1.4% (n=2), enteral feeding by 6.3% (n=9), and diapers by 60.6% (n=86). Sedative use was recorded in 19.7% (n=28) of participants. Using the full sample (N=142) as the denominator for dementia types, Alzheimer disease was the most common (n=81, 57%), followed by vascular dementia (n=15, 10.6%), Lewy body dementia (n=3, 2.1%), and mixed dementia (n=1, 0.7%; Table 1).

**Table 1:** Participants’ general and clinical characteristics (N=142).

Variable			Participants
Age (years), mean (SD)			80.92 (7.26)
**Sex, n (%)**			
	Male	59 (41.5)	
	Female	83 (58.5)	
Smoking, n (%)			3 (2.1)
Alcohol, n (%)			0 (0)
**Residence before admission, n (%)**			
	House	93 (65.5)	
	Hospital	41 (28.9)	
	Others	8 (5.6)	
BMI (kg/m^2^), mean (SD)			21.97 (3.44)
Charlson Comorbidity Index score, mean (SD)			5.54 (1.81)
K-MMSE^a^, mean (SD)			10.89 (7.35)
Number of medications, mean (SD)			7.50 (4.81)
**Present disease (multiple responses permitted), n (%)**			
	Hypertension	76 (53.5)	
	Diabetes	37 (26.1)	
	Degenerative arthritis	3 (2.1)	
	Cancer	77 (54.2)	
	COVID-19	2 (1.4)	
	Stroke	26 (18.3)	
	History of brain damage	15 (10.6)	
	Vision disturbances or hearing loss	1 (0.7)	
	Dehydration (BUN^b^>18 mg/dL)	7 (4.9)	
	Malnutrition	56 (39.4)	
	Electrolyte imbalance	38 (26.8)	
	Surgery	11 (7.7)	
	Infection	56 (39.4)	
	Pain	28 (19.7)	
	Insomnia	12 (8.5)	
	Restraints	5 (3.5)	
	Immobility	36 (25.4)	
	Falls	26 (18.3)	
	Pressure ulcer	24 (16.9)	
	Analgesic use	23 (16.2)	
	Delirium-related medication (haloperidol)	14 (9.9)	
	Transfusion	1 (0.7)	
	Indwelling urinary catheterization	2 (1.4)	
	Enteral feeding	9 (6.3)	
	Diaper use	86 (60.6)	
	Sedative use	28 (19.7)	
	Dementia	100 (70.4)	
**Type of dementia, n (%)**			
	Alzheimer	81 (57)	
	Vascular	15 (10.6)	
	Mixed	1 (0.7)	
	Lewy body	3 (2.1)	

^a^K-MMSE: Korean version of the Mini-Mental State Examination.

^b^BUN: blood urea nitrogen.

### Delirium Occurrence and Motor Subtypes

During the 4-week assessment period, delirium was identified in 38.7% (55/142) of patients, including 41% (41/100) of patients with dementia and 33.3% (14/42) of patients with MCI. Among patients with delirium, the hypoactive type was most common (33/55, 60%), followed by hyperactive (18/55, 32.7%) and mixed (4/55, 7.3%) types. In the dementia group, hypoactive, hyperactive, and mixed delirium accounted for 65.9% (27/41), 26.8% (11/41), and 7.3% (3/41) of patients, respectively. In the MCI group, the corresponding proportions were 42.9% (6/14), 50% (7/14), and 7.1% (1/14; Table 2).

**Table 2:** Delirium occurrence and motor subtypes.

Variable			Group, n (%)							*P* value	
			Overall (N=142)		Dementia (n=100)		MCI^a^ (n=42)				
**Delirium**											.45
	Yes	55 (38.7)		41 (41)		14 (33.3)					
**Motor subtype among patients with delirium**											.25
	Hypoactive	33 (60)		27 (65.9)		6 (42.9)					
	Hyperactive	18 (32.7)		11 (26.8)		7 (50)					
	Mixed	4 (7.3)		3 (7.3)		1 (7.1)					

^a^MCI: mild cognitive impairment.

### Comparison of Variables by Cognitive Status and Delirium Occurrence

The 4 groups differed in sex (*P*=.006), admission source (*P*<.001), CCI score (*P*=.005), K-MMSE score (*P*<.001), number of medications (*P*<.001), cancer (*P*<.001), history of COVID-19 (*P*=.047), infection (*P*=.03), immobility (*P*<.001), analgesic use (*P*=.03), urinary catheterization (*P*=.009), and diaper use (*P*<.001; Table 3). Haloperidol use also differed across groups (*P*<.001) and was recorded only in patients with delirium.

**Table 3:** Comparison of variables by cognitive status and delirium occurrence (N=142).

Variable			Dementia					Mild cognitive impairment					*F* test (*df*)				Chi-square (*df*)				*P* value	
			DSD^a^ (n=41)		Non-DSD^b^ (n=59)		MCI-D^c^ (n=14)			Non-MCI-D^d^ (n=28)												
Age (years), mean (SD)			81.37 (7.95)		80.59 (7.22)		80.79 (7.03)			81.04 (6.75)		0.093 (3, 138)				—^e^				.96		
**Sex, n (%)**														—				12.5 (3)				.006
	Male	11 (26.8)		31 (52.5)		2 (14.3)			15 (53.6)													
	Female	30 (73.2)		28 (47.5)		12 (85.7)			13 (46.4)													
**Smoking, n (%)**														—				—				.85
	Yes	1 (2.4)		1 (1.7)		0 (0)			1 (3.6)													
**Alcohol consumption, n (%)**														—				—				—
	Yes	0 (0)		0 (0)		0 (0)			0 (0)													
**Residence before admission, n (%)**														—				—				<.001
	Home	22 (53.7)		33 (55.9)		12 (85.7)			26 (92.9)													
	Hospital	17 (41.5)		22 (37.3)		2 (14.3)			0 (0)													
	Others	2 (4.9)		4 (6.8)		0 (0)			2 (7.1)													
BMI (kg/m^2^), mean (SD)			21.89 (3.77)		21.43 (3.24)		23.29 (3.20)			22.56 (3.35)		1.468 (3, 138)				—				.23		
CCI^f^ score, mean (SD)			5.93 (2.00)		5.81 (1.58)		5.36 (2.02)			4.50 (1.53)		4.503 (3, 138)				—				.005		
K-MMSE^g^ score, mean (SD)			7.05 (4.63)		7.00 (5.23)		19.50 (1.70)			20.43 (1.32)		92.558 (3, 138)				—				<.001		
Number of medications, mean (SD)			10.24 (3.20)		9.37 (2.97)		3.29 (4.66)			1.64 (3.51)		51.634 (3, 138)				—				<.001		
**Hypertension, n (%)**														—				1.5 (3)				.68
	Yes	24 (58.5)		28 (47.5)		8 (57.1)			16 (57.1)													
**Diabetes, n (%)**														—				2.9 (3)				.40
	Yes	12 (29.3)		16 (27.1)		5 (35.7)			4 (14.3)													
**Degenerative arthritis, n (%)**														—				—				.50
	Yes	1 (2.4)		1 (1.7)		1 (7.1)			0 (0)													
**Cancer, n (%)**														—				28.3 (3)				<.001
	Yes	30 (73.2)		38 (64.4)		5 (35.7)			4 (14.3)													
**COVID-19, n (%)**														—				—				.047
	Yes	0 (0)		0 (0)		0 (0)			2 (7.1)													
**Stroke, n (%)**														—				6.9 (3)				.08
	Yes	6 (14.6)		16 (27.1)		0 (0)			4 (14.3)													
**History of brain damage, n (%)**														—				—				.60
	Yes	5 (12.2)		7 (11.9)		2 (14.3)			1 (3.6)													
**Vision or hearing impairment, n (%)**														—				—				>.99
	Yes	0 (0)		1 (1.7)		0 (0)			0 (0)													
**Dehydration (BUN** ^h^ **>18 mg/dL), n (%)**														—				—				.20
	Yes	3 (7.3)		1 (1.7)		0 (0)			3 (10.7)													
**Malnutrition, n (%)**														—				2.0 (3)				.58
	Yes	15 (36.6)		27 (45.8)		4 (28.6)			10 (35.7)													
**Electrolyte imbalance, n (%)**														—				3.5 (3)				.32
	Yes	15 (36.6)		12 (20.3)		3 (21.4)			8 (28.6)													
**Surgery, n (%)**														—				—				.15
	Yes	4 (9.8)		3 (5.1)		3 (21.4)			1 (3.6)													
**Infection, n (%)**														—				8.8 (3)				.03
	Yes	10 (24.4)		26 (44.1)		4 (28.6)			16 (57.1)													
**Pain, n (%)**														—				5.9 (3)				.12
	Yes	11 (26.8)		6 (10.2)		4 (28.6)			7 (25)													
**Insomnia, n (%)**														—				—				.18
	Yes	3 (7.3)		7 (11.9)		2 (14.3)			0 (0)													
**Restraints, n (%)**														—				—				.56
	Yes	1 (2.4)		4 (6.8)		0 (0)			0 (0)													
**Immobility, n (%)**														—				17.0 (3)				<.001
	Yes	19 (46.3)		13 (22)		3 (21.4)			1 (3.6)													
**Falls, n (%)**														—				7.0 (3)				.07
	Yes	12 (29.3)		7 (11.9)		4 (28.6)			3 (10.7)													
**Pressure ulcer, n (%)**														—				—				.30
	Yes	11 (26.8)		8 (13.6)		2 (14.3)			3 (10.7)													
**Analgesic use, n (%)**														—				—				.03
	Yes	10 (24.4)		4 (6.8)		4 (28.6)			5 (17.9)													
**Delirium-related medication (haloperidol), n (%)**														—				—				<.001
	Yes	6 (14.6)		0 (0)		8 (57.1)			0 (0)													
**Transfusion, n (%)**														—				—				.10
	Yes	0 (0)		0 (0)		1 (7.1)			0 (0)													
**Indwelling urinary catheterization, n (%)**														—				—				.009
	Yes	0 (0)		0 (0)		2 (14.3)			0 (0)													
**Enteral feeding, n (%)**														—				—				.31
	Yes	4 (9.8)		5 (8.5)		0 (0)			0 (0)													
**Diaper use, n (%)**														—				42.3 (3)				<.001
	Yes	31 (75.6)		44 (74.6)		9 (64.3)			2 (7.1)													
**Sedative use, n (%)**														—				5.4 (3)				.14
	Yes	12 (29.3)		12 (20.3)		2 (14.3)			2 (7.1)													

^a^DSD: delirium superimposed on dementia.

^b^Non-DSD: dementia without delirium.

^c^MCI-D: mild cognitive impairment with delirium.

^d^Non-MCI-D: mild cognitive impairment without delirium.

^e^ —: not applicable. *F* statistics are reported for ANOVA and *χ*² statistics for Pearson chi-square tests. Neither statistic applies to Fisher-Freeman-Halton exact tests. No test was performed for alcohol consumption because all participants reported no alcohol use.

^f^CCI: Charlson Comorbidity Index.

^g^K-MMSE: Korean version of the Mini-Mental State Examination.

^h^BUN: blood urea nitrogen.

### Factors Associated With Delirium

Overall, female sex (adjusted OR [aOR] 4.344, 95% CI 1.794-10.515; *P*=.001), immobility (aOR 4.953, 95% CI 1.857-13.205; *P*=.001), and analgesic use (aOR 3.641, 95% CI 1.262-10.501; *P*=.02) were positively associated with delirium, whereas infection was inversely associated with delirium (aOR 0.251, 95% CI 0.101-0.623; *P*=.003). Age (*P*=.69), CCI score (*P*=.69), and K-MMSE score (*P*=.48) were not significantly associated with delirium. The maximum variance inflation factor was 1.22. The Hosmer-Lemeshow test showed no evidence of poor fit (*χ*^²^₈=7.1; *P*=.53). In the dementia group, female sex, immobility, analgesic use, and pain were positively associated with delirium, and infection was inversely associated with delirium. In the MCI group, diaper use was associated with delirium (aOR 20.327, 95% CI 3.005-137.500; *P*=.002; Table 4).

**Table 4:** Factors associated with deliriuma.

Variable	Overall (N=142)		Dementia (n=100)		MCI^b^ (n=42)	
	aOR^c^ (95% CI)	*P* value	aOR (95% CI)	*P* value	aOR (95% CI)	*P* value
Age	0.988 (0.931-1.048)	.69	—^d^	—	—	—
Female sex	4.344 (1.794-10.515)	.001	4.013 (1.410-11.423)	.009	5.552 (0.767-40.187)	.09
CCI^e^ score	0.952 (0.749-1.211)	.69	—	—	—	—
K-MMSE^f^ score	0.980 (0.925-1.037)	.48	—	—	—	—
Infection	0.251 (0.101-0.623)	.003	0.166 (0.052-0.537)	.003	—	—
Immobility	4.953 (1.857-13.205)	.001	6.302 (1.982-20.041)	.002	—	—
Analgesic use	3.641 (1.262-10.501)	.02	5.345 (1.106-25.818)	.04	—	—
Falls	—	—	2.252 (0.605-8.387)	.23	—	—
Pain	—	—	6.073 (1.326-27.818)	.02	—	—
Diaper use	—	—	—	—	20.327 (3.005-137.500)	.002

^a^Reference categories were male sex and absence of each binary characteristic.

^b^MCI: mild cognitive impairment.

^c^aOR: adjusted odds ratio.

^d^Not applicable; the variable was not included in the corresponding regression model.

^e^CCI: Charlson Comorbidity Index.

^f^K-MMSE: Korean version of the Mini-Mental State Examination.

## Discussion

This study examined delirium occurrence and associated factors among patients with dementia or MCI in LTC hospitals. Delirium was identified in 38.7% (55/142) of patients, and hypoactive delirium was the most common subtype. Female sex, immobility, and analgesic use were positively associated with delirium overall, whereas infection was inversely associated with delirium.

Delirium was identified in 38.7% of patients, compared with 48% in the Korean LTC hospital study by Moon and Park [3], which used the same assessment instrument. Their study included patients with and without dementia and assessed delirium for 3 months, whereas our study focused on patients with dementia or MCI over 4 weeks. Differences in populations and assessment periods also contribute to the wide range of prevalence estimates in residential care (1%-70.3%) [14]. Despite these differences, delirium affected more than one-third of our participants, supporting the need for regular assessment in patients receiving care for cognitive impairment.

Hypoactive delirium was the most common subtype overall (33/55, 60%) and in patients with dementia (27/41, 65.9%), whereas hyperactive delirium was most common in patients with MCI (7/14, 50%). The predominance of hypoactive delirium is consistent with the ICU findings of la Cour et al [21], where this subtype accounted for approximately 50.3% of cases. However, mixed delirium predominated in the LTC hospital study by Moon and Park [3]. Ghezzi et al [22] found poorer cognition in patients with hypoactive than hyperactive delirium, although the effect size was small. These findings highlight the relevance of cognitive status when interpreting motor patterns. Hypoactive delirium may be overlooked when patients appear quiet [9], making observation of changes in activity and attention important [23]. In patients with dementia, nurses need to compare these changes with usual behavior. In patients with MCI, both increased and reduced activity require attention, as hypoactive delirium still accounted for 42.9% (6/14) of patients with MCI who developed delirium.

Female sex, immobility, and analgesic use remained associated with delirium after adjustment for age, CCI score, and K-MMSE score. Patients with dementia may have difficulty expressing pain [24,25], and pain has been associated with delirium in this population [26]. A meta-analysis also identified an association between pain and delirium among older hospital inpatients, although most included studies involved surgical patients [27]. Our finding for analgesic use therefore needs to be interpreted alongside pain and the reasons for treatment. Mobility also warrants attention, as physical dysfunction has been linked to delirium [28], and immobility was associated with delirium in the univariable analysis of older patients undergoing cholecystectomy [29]. Together, these findings support assessing pain in ways suited to patients’ communication abilities and considering mobility, pain relief, and changes in attention during daily care.

Infection was inversely associated with delirium in our study. Associations between systemic infection and delirium in residential care have also been inconsistent or nonsignificant [14]. Our infection variable included physician diagnosis, laboratory evidence, or antibiotic use, and its timing relative to delirium was unknown. The inverse association should therefore not be interpreted as a protective effect of infection.

Diaper use was associated with delirium in the MCI group, with use recorded in 64.3% (9/14) of patients with delirium and 7.1% (2/28) without delirium. The association remained after adjustment for sex, but the wide CI indicates limited precision. Diaper use may reflect a need for assistance with continence, mobility, or toileting, although these needs were not established by our data. Clinically, the finding draws attention to the circumstances of diaper use and the patient’s functional status. Documenting the reasons for diaper use and changes in toileting ability alongside cognitive and behavioral changes may help nurses assess these care needs. Future studies should examine these factors before delirium develops to clarify the observed association.

Delirium was identified in one-third of patients with MCI, indicating a need for assessment, as well as in patients with dementia. Sabbagh et al [30] emphasized linking MCI recognition with further assessment and appropriate care in primary care. In acute care hospitals, gaps remain in cognitive assessment, documentation, and communication with patients and caregivers [31]. These findings are relevant to continuity of cognitive care in LTC hospitals, where nurses can document usual cognitive and functional status, share changes during handover, and consult familiar caregivers when establishing the patient’s baseline. Regular delirium assessment, together with attention to mobility, pain, and toileting needs, provides a focus for nursing care. The effectiveness of preventive care based on these findings requires evaluation in LTC hospitals, where intervention evidence remains limited [12].

This study has several limitations. First, data were collected during the COVID-19 pandemic, when masks and changes in the care environment and social contact may have affected delirium assessment and occurrence. Assessment completeness was not quantified, and formal interrater reliability was not evaluated during data collection. Symptoms of dementia and delirium can also overlap, complicating assessment in patients with moderate to severe dementia despite training [32]. Second, the timing of clinical conditions and care practices relative to delirium was unknown, precluding causal interpretation. Details of sedative use and the interval and type of prior surgery were unavailable. Third, the sample size was calculated for multiple linear regression rather than the logistic models used. The small cognitive groups, particularly the MCI group, limited estimate precision. Fourth, pain assessment included medication use, and infection assessment included antibiotic use, limiting the separation of symptoms from treatment-related associations. We did not adjust for multiple comparisons. Finally, recruitment from 2 LTC hospitals limits generalizability to other settings and patients without cognitive impairment. Larger longitudinal studies are needed to confirm these associations.

Delirium was common among patients with dementia or MCI in LTC hospitals, and hypoactive delirium was the most common subtype overall. Female sex, immobility, and analgesic use were positively associated with delirium, whereas infection was inversely associated. These findings support regular delirium assessment, with attention to mobility and pain-related care needs. Further longitudinal studies are needed to clarify these associations and the findings in each cognitive group.

## Appendix

Multimedia Appendix 1 STROBE checklist.

## Abbreviations

aOR: adjusted odds ratio
CCI: Charlson Comorbidity Index
DSD: delirium superimposed on dementia
ICU: intensive care unit
K-MMSE: Korean version of the Mini-Mental State Examination
LTC: long-term care
MCI: mild cognitive impairment
MCI-D: mild cognitive impairment with delirium
Non-DSD: dementia without delirium
Non-MCI-D: mild cognitive impairment without delirium
S-CAM: Short Confusion Assessment Method
STROBE: Strengthening the Reporting of Observational Studies in Epidemiology

## Data Availability

The data are not publicly available because they contain sensitive patient information. Deidentified data may be requested from the corresponding author, subject to consent and institutional approval.
